# Editorial: Nanotechnology facilitated photo (electrochemcial) biosensors

**DOI:** 10.3389/fbioe.2023.1136565

**Published:** 2023-01-17

**Authors:** Qing Liu, Dong Liu, Mario A. Alpuche-Aviles, Wei Ma

**Affiliations:** ^1^ School of Chemistry and Chemical Engineering, Shandong University of Technology, Zibo, China; ^2^ Key Laboratory of Modern Agricultural Equipment and Technology, School of Agricultural Engineering, Jiangsu University, Zhenjiang, China; ^3^ Department of Chemistry, University of Nevada, Reno, NV, United States; ^4^ Feringa Nobel Prize Scientist Joint Research Center, Frontiers Science Center for Materiobiology and Dynamic Chemistry, Key Laboratory for Advanced Materials and Joint International Research Laboratory of Precision Chemistry and Molecular Engineering, School of Chemistry and Molecular Engineering, East China University of Science and Technology, Shanghai, China

**Keywords:** electrochemical, photoelectrochemcial, biosensor, nanotechnology, nanomaterial

Photoelectrochemical biosensor is a new class of analytical method for biosensing based on the photoelectrochemical properties of nanomaterials (Zhao et al., 2015). Recently, the rapid development of nanomaterials has opened up new avenues for their use in photoelectrochemical applications owing to their special properties in optical, electrical, and mechanical strength. These unique properties of nanomaterials at the nanoscale dimension compared to their bulk counterparts have attracted much attentions for photoelectrochemical sensing. On the one hand, the introduction of nanotechnology has led to the development of novel biosensors and biosensing mechanisms, as well as the improvement in sensitivity and selectivity of the existing biosensors in the various field. On the other hand, the nanoscale dimension of materials further assists the development of biosensors for rapid and simple detection *in vivo* as well as the ability to detect single-biomolecules (Dai et al., 2022). Much efforts have been devoted for design and fabrication of photoelectrochemical biosensors over the past decades. The photoelectrochemical biosensors with highly sensitive and selective performances have been widely applied in healthcare, environment, and food areas (Qiu et al., 2020). In order to gain new knowledge in the field of photoelectrochemical biosensors, especially based on nanotechnology, we have been working on the Research Topic “Nanotechnology facilitated photo (electrochemcial) biosensors.” It includes four articles (one review paper and three original research articles) written by Chinese scientists. We hereby express our sincere gratitude for the efforts and contributions of all those involved in this Research Topic.

Detection of disease biomarkers in clinical samples is crucial for the early diagnosis and the treatment of disease because biomarker can reflect the progression and prognosis of tumor. With the increasing demand for ultrasensitive detection, there is an urgent need to develop novel sensors for biomarker analysis (Shi et al., 2022). In recent years, the newly developed photo (electrochemcial) biosensors have become one of the most promising method for tumor biomarker detection with high specificity and sensitivity. More importantly, the combination of photo(electrochemcial) techniques with biosensing strategies have been proved to be more advantageous than conventional sensing methods due to the more powerful signal amplification systems. In this Research Topic, Cao et al. designed a novel electrochemical immunosensor based on PdAgPt/MoS_2_ for the ultrasensitive detection of CA242, which has been used in the diagnosis of colorectal cancer, pancreatic cancer, gastric cancer and other cancers. In this study, the prepared PdAgPt nanocomposites were loaded onto the surface of MoS_2_ with large surface area as signal amplification platform. Moreover, the electrochemical immunosensor of peroxisase-like activity constructed by PdAgPt nanoparticles and molybdenum disulfide can co-amplify the current signal. Due to the specific binding of antigen and antibody, CA242 can be quantified according to the decreased magnitude of the current signal. Accordingly, this label-free electrochemical immunosensor of CA242 has a wide detection range from 1 × 10^–4^ U/mL to 1 × 10^2^ U/mL and a low detection limit (LOD, 3.43 × 10^–5^ U/mL). Therefore, the label-free electrochemical immunosensor in this study performed well in the evaluation of repeatability, selectivity and stability for the detection of clinical biomarkers.

Conventional electrochemical detection of biomarker are mainly used to modify the surface of the induction electrode of immunosensors. However, the pretreatment of electrode (such as polishing) makes the clinical detection to be cumbersome. As well-known, screen-printed electrodes are excellent electrochemical sensor elements that do not require cumbersome pretreatment and have abilities with low cost, small size, flexible design, and easily integration with other equipments (e.g., microfluidic device). Wang et al. reported a broad-range disposable electrochemical biosensor based on screen-printed carbon electrodes for detection of human noroviruses. In this work, an electrochemical biosensor for sensitive and fast detection of HuNoVs was constructed. Gold nanoparticles and protein-A were applied on the screen-printed carbon electrode surface for enhancement of the electrical signals and the linkage of antibodies with a fixed orientation, respectively. A monoclonal antibody against the S domain protein of the viral capsid was further immobilized on the electrode to bind HuNoVs specifically. The binding of viral capsid to the coated monoclonal antibody resulted in the reduction of conductivity (current) measured by cyclic voltammetry and differential pulse voltammetry. The detection limitation of Genogroup I.1 viral capsid and Genogroup II.4 viral capsid was 0.37 ng/mL (≈1.93 × 107 HuNoVs/mL) and 0.22 ng/mL (≈1.15 × 107 HuNoVs/mL), respectively. This disposable electrochemical biosensor was a good candidate for rapid detection of different genogroup and genotype HuNoVs.

Since the traditional diagnostic tests need to analyze the clinic sample in a laboratory and obtain the results after hours and even several days, point-of-care testing that occurs near to the patient bedside and requires rapid turnaround time of test results is urgently needed for clinical decision making. For example, the real-time and convenient detection of glucose in biofiuids displays great potential not only in continuous, long-term monitoring of various kinds of diseases including diabetes. Traditional glucose testing is detecting glucose in blood and is invasive, which cannot be continuous and is discomfort for the users. Consequently, wearable glucose sensors toward continuous point-of-care glucose testing in biofiuids have attracted great attention, and the trend of glucose testing is from invasive to non-invasive. Zhang et al. reviewed the wearable point-of-care glucose sensors for the detection of different biofiuids including blood, sweat, saliva, tears, and the future trend of development is prospected. Wang et al. reported a multichannel microelectrode array for real-time detection of local field potential and action potential (spike) in the suprachiasmatic nucleus in induced torpor mice. Platinum nanoparticles and Nafion membrane modified multichannel microelectrode array has a lower impedance (16.58 ± 3.93 kΩ) and higher signal-to-noise ratio (S/N = 6.1). They found that from torpor to arousal, the proportion of theta frequency bands of LFPs increased and spike firing rates rapidly increased. These results could be characteristic information of arousal, which was supported by the microscopic neural activity promoting arousal in mice. Multichannel microelectrode array displayed real-time dynamic changes of neuronal activities in the suprachiasmatic nucleus, which was more helpful to analyze and understand neural mechanisms of torpor and arousal.

Despite the development of nanotechnology facilitated photoelectrochemical biosensors by leaps and bounds, there are still some drawbacks to be further improved in the following aspects: 1) It is necessary to develop advanced manufacturing processes for improving the poor stability and reproducibility of photoelectrochemical biosensors in practical applications; 2) The controlled preparation of functional nanomaterials can provide a new prospect for the further development of nanotechnology facilitated photoelectrochemical biosensing; 3) The development and design of high-performance integrated substitutable electronics detection components and portable devices can realize the real-time monitoring of complex signals. In order to further enhance the detection performance of photoelectrochemical biosensors and expand their application range, we offer the following take-home messages for consideration in future research and development. Firstly, the effective interface nanofabrication plays a critical role in the construction of high-performance biosensing platforms for detecting target molecules through various analytical principles. Secondly, exploiting advanced functional materials with rich nanostructures can amplify biorecognition events and accelerate the signal transduction, resulting in highly sensitive biosensing. Thirdly, designing signal strategies based on specific biological recognition components such as antibodies, nucleic acids and enzymes can also improve a lot on the analytical performance of photoelectrochemical biosensors. Finally, by greatly reducing background signals, the photoelectrochemical method becomes very sensitive with the target bioanalytes. Compared to conventional electrochemical techniques, using light as excitation source and photocurrent as recognition signal contributes to lower background noise and higher sensitivity. In conclusion, the Research Topic of papers in this Research Topic “Nanotechnology facilitated photo (electrochemcial) biosensors” covers a broad area of research from fundamental understanding of the role of photoelectrochemical biosensing. With these rapid progresses in the efuture, we believe that photoelectrochemical analysis will certainly play a bigger role for biosensing.
